# Peptide-Based Nanocomplexes Enable Transferrin-Mediated Uptake and p53-Driven Antitumor Activity in 2D and 3D Glioblastoma Models

**DOI:** 10.3390/ijms27156857

**Published:** 2026-07-30

**Authors:** Leonor M. Castro, Ana R. Neves, Eric Vivès, Prisca Boisguérin, Ângela Sousa, Diana Costa

**Affiliations:** 1 RISE-Health, Department of Medical Sciences, Faculty of Health Sciences, University of Beira Interior, Av. Infante D. Henrique, 6200-506 Covilhã, Portugal; leonor.m.castro@ubi.pt (L.M.C.); a34643@ubi.pt (A.R.N.); angela@fcsaude.ubi.pt (Â.S.); 2 PhyMedExp, University of Montpellier, INSERM, CNRS, 34295 Montpellier, France; eric.vives@umontpellier.fr (E.V.); prisca.boisguerin@inserm.fr (P.B.)

**Keywords:** design of experiments, drug/gene co-delivery, glioblastoma, peptide-based nanocarriers, spheroid model, targeted peptide-based nanosystems, *TP53* gene

## Abstract

Glioblastoma (GB), the most prevalent and aggressive brain tumor, remains one of the most lethal challenges in modern oncology. Standard therapy remains largely ineffective, mainly due to limited therapeutic penetration across the blood–brain barrier (BBB) and adaptive tumor resistance. Consequently, there is an urgent need for innovative strategies to enhance therapeutic precision and efficacy. To address these limitations, we engineered a targeted peptide-based co-delivery system using the WRAP5 cell-penetrating peptide functionalized with a transferrin receptor (TfR)-targeting T7 peptide (sequence: HAIYPRH), enabling simultaneous delivery of temozolomide (TMZ) and a p53-encoding plasmid DNA. The resulting peptide-based TMZ/p53 nanocomplexes exhibited favorable physicochemical properties, enhanced TfR-mediated cellular uptake, and targeted antitumor activity mediated through p53-induced apoptosis in two-dimensional (2D) U87 MG cell cultures. To better reproduce the structural and cellular complexity of the tumor microenvironment, a three-dimensional U87 MG spheroid model was established and optimized using a Design of Experiments (DoE) approach to improve reproducibility and physiological relevance. The developed WRAP5-based nanocomplexes induced a significant dose-dependent inhibition of growth and morphological alterations in the U87 MG spheroid model, accompanied by deep penetration and cell death throughout the spheroid. Collectively, these findings highlight the potential of this targeted and tailored nanosystem to enhance cellular transfection, enable drug/gene co-delivery, restore p53 function, and promote apoptosis, representing a promising therapeutic strategy for GB treatment.

## 1. Introduction

Glioblastoma (GB), classified by the World Health Organization (WHO) (2021) as a grade 4 isocitrate dehydrogenase (IDH) wild-type astrocytoma, is the most prevalent and lethal brain tumor in adults [1,2,3]. The standard-of-care Stupp protocol comprises surgery, radiotherapy, and temozolomide (TMZ) chemotherapy. TMZ, an oral alkylating agent derived from dacarbazine, is typically administered following surgical resection and radiotherapy. However, its efficacy is often limited by poor drug penetration and the development of tumor resistance. The treatment extends median survival to approximately 15 months, with only 6% of patients surviving five years. Therapeutic failure is driven by a combination of factors, including tumor heterogeneity, infiltrative growth, *TP53*-associated chemoresistance, and constraints imposed by the blood–brain barrier (BBB), which limit drug penetration into the tumor [1,4,5,6]. This highlights the urgent need for innovative therapeutic strategies. Local delivery of drug-loaded nanosystems via intracranial injection directly to the tumor bed following resection appears to be a promising strategy for overcoming the challenges associated with BBB penetration and bloodstream removal. This approach has shown promising results that this route of administration enhances drug retention at the tumor site and improves therapeutic efficacy [7,8,9].

Gene therapy, which relies on the use of genetic material to prevent or treat specific diseases, is a potential tool for managing GB treatment because of its unique therapeutic advantages [10,11]. Approaches aimed at re-establishing p53 function represent a particularly compelling therapeutic avenue [1,12,13,14]. The *TP53* gene is a central determinant of GB pathobiology, with mutations observed in up to 30% of primary and 70% of secondary tumors. As a critical tumor suppressor and “guardian of the genome,” p53 orchestrates key cellular processes, including cell cycle regulation, DNA repair, and apoptosis [15,16]. Functional disruption is frequently driven by alterations in the p53–ARF–MDM2 axis. These alterations result in the loss of genomic control, enabling unchecked proliferation and invasive behavior. Furthermore, they contribute to the pronounced resistance to TMZ, the most effective chemotherapeutic agent currently available for GB. Notably, mutant p53 can acquire gain-of-function properties, such as the formation of amyloid-like aggregates, which further exacerbate tumor aggressiveness, stemness, and chemoresistance [17,18,19]. Overall, *TP53* dysregulation is strongly associated with poor clinical outcomes and represents a compelling target for the development of next-generation therapeutic strategies for GB.

The integration of chemotherapy and gene therapy is becoming increasingly prominent in cancer treatment [20,21,22]. The co-delivery of multiple therapeutic agents enhances tumor suppression while allowing dose reduction, driven by synergistic interactions arising from their combined mechanisms of action. The dual delivery of TMZ and the *TP53* gene leads to tumor inhibition, reduced TMZ dosage, and synergistic killing of GB cells [21,23,24]. Specifically, p53 upregulation enhances GB sensitivity to TMZ by suppressing O6-methylguanine-DNA methyltransferase (MGMT), a key DNA repair enzyme that counteracts TMZ-induced damage. p53 also modulates DNA methyltransferases (DNMTs), inducing apoptosis and G1 arrest, while regulating DNA repair and senescence pathways [25,26,27,28]. Consequently, p53 supplementation may be an effective strategy for overcoming TMZ resistance [21].

Achieving effective dual delivery requires the development of nanocarrier platforms capable of co-encapsulation or co-complexing, protection, and controlled delivery of both drugs and genetic cargoes. Therefore, non-viral delivery systems have emerged as useful tools to facilitate cellular internalization and targeted intracellular delivery, ultimately enhancing the outcomes of cancer therapy [29,30]. Cell-penetrating peptides (CPPs), particularly members of the WRAP family, efficiently transport biomolecules across cell membranes [1,21,31]. In GB, CPPs can address key therapeutic bottlenecks, including limited BBB permeability, inadequate tumor targeting, and reduced TMZ cytotoxicity [32,33,34]. Surface functionalization with the HAIYPRH (T7) heptapeptide provides a precise strategy to exploit the overexpression of the transferrin receptor (TfR) within the brain tumor microenvironment in a non-competitive manner to endogenous transferrin. Given that TfR is expressed on BBB endothelial cells and is significantly overexpressed (up to 100-fold) in GB cells compared with healthy brain tissue, it serves as a promising dual-targeting strategy for brain penetration and tumor targeting [35,36,37,38,39].

Three-dimensional (3D) cell culture systems, such as tumor spheroids, have emerged as indispensable tools for improving the predictive power of preclinical research [40,41]. Unlike two-dimensional (2D) monolayers, spheroids develop spatial heterogeneity, including proliferative outer zones and hypoxic/necrotic cores. These features more accurately mimic the diffusion gradients and therapeutic resistance observed in solid tumors [42,43,44]. Among the various fabrication strategies, the liquid overlay technique (LOT) using agarose-coated wells provides a reproducible, biocompatible, and cost-effective scaffold-free platform for generating uniform multicellular aggregates [45,46,47].

The application of Design of Experiments (DoE) has become a vital statistical methodology in pharmaceutical development. By systematically and simultaneously varying multiple input factors, DoE enables the identification and quantification of complex cause-and-effect relationships that conventional one-factor-at-a-time (OFAT) approaches typically overlook [48,49,50,51,52]. Response surface designs, specifically central composite designs (CCD), can be used to model non-linear relationships and identify optimal operating regions [49,51,53,54].

In the present study, a T7-functionalized WRAP5 nanosystem was developed for the co-delivery of TMZ and the *TP53* gene to enhance therapeutic response in GB. By leveraging a DoE framework, we optimized the formation and standardization of a 3D GB spheroid model (LOT), establishing a robust and physiologically relevant platform to investigate the impact of this peptide-based drug/gene strategy on tumor viability and structural integrity. The results obtained from the 2D and 3D GB models demonstrate that the T7-functionalized TMZ/p53 nanocomplexes mediated cellular uptake, p53 expression, caspase induction, and tumor inhibition in U87 MG cells. This nanoplatform represents a potential tool for precise, coordinated, and more effective GB therapy, facilitating clinical translation.

## 2. Results

### 2.1. Development of TMZ-WRAP5/T7/pDNA Complexes

The assembly of TMZ-WRAP5/T7/pDNA nanocomplexes was achieved by controlled dropwise addition of the TMZ-WRAP5 conjugate to a solution containing pDNA and the T7-targeting peptide under vortexing (Figure 1A). This co-precipitation method facilitated the electrostatic condensation of anionic pDNA by the cationic peptide components.

To determine the optimal nitrogen-to-phosphate (N/P) ratio for pDNA complexation, agarose gel electrophoresis was employed (Figure 2). At lower N/P ratios (0.1 and 0.2), a distinct band corresponding to uncomplexed pDNA was observed, indicating insufficient complexation (Figure S1, available in the Supplementary Materials (SM)). As the N/P ratio increased to 0.5, the intensity of the free pDNA band decreased significantly, resulting in complete complexation at an N/P ratio of 1. These findings confirmed the formation of stable nanocomplexes through total pDNA complexation (Figure 2A). Although the T7 peptide carries one additional positive charge, control assays demonstrated that T7 alone provided significantly lower complexation efficiency compared with the complete TMZ-WRAP5/T7/pDNA nanocomplex or non-targeted complexes (Figure 2B). However, a slight increase in pDNA retention in the complexes was observed in the formulations containing T7, particularly at an N/P ratio of 0.5.

Spectrophotometric analysis (330 nm) of the supernatants was performed to evaluate TMZ loading efficiency. The results reveal high drug retention within the nanocomplexes, as presented in Table 1. Incorporation efficiencies remained high across the N/P ratios tested, with no significant variance between N/P 0.5 (88.7%) and N/P 1 (86.6%). Notably, the TMZ-WRAP5/T7/pDNA architecture yielded a 2.3-fold increase in TMZ loading capacity compared with the alternative formulation approach (Table S1), highlighting the superior payload-carrying capacity of the conjugated peptide system.

#### 2.1.1. Physicochemical Characterization

After formulation, the nanocomplexes were thoroughly characterized to determine particle morphology, size, size distribution, and surface charge. Scanning electron microscopy (SEM) confirmed that both targeted and non-targeted complexes formed spherical, non-aggregated particles with no obvious agglomeration, as displayed in Figure 3A,B. Dynamic Light Scattering (DLS) analysis (Figure 3C–E) revealed a clear correlation between the N/P ratio and particle size; specifically, higher N/P ratios promoted more efficient pDNA complexation, resulting in smaller hydrodynamic diameters. For the TMZ-WRAP5/T7/pDNA nanosystem, the mean diameter decreased from 340 ± 4.76 nm at N/P 0.1 to 213 ± 3.90 nm at N/P 1. In comparison, T7/pDNA complexes exhibited inferior condensation, remaining larger even at higher ratios (274 ± 2.42 nm at N/P 1) (Figure 3C). The same trend was observed for the zeta potential, showing a progressive increase in the surface charge with increasing peptide concentration. The charge of the TMZ-WRAP5/T7/pDNA system underwent charge inversion, shifting from −9.81 ± 0.88 mV (N/P 0.1) to +11.39 ± 0.80 mV (N/P 1), confirming the dominant role of WRAP5 in modulating surface electrostatic interactions compared with T7 alone (Figure 3E). The polydispersity index (PdI) values indicated moderately broad size distributions (0.430–0.492 ± 0.02) for the targeted system, while the non-targeted formulation (TMZ-WRAP5/pDNA) exhibited narrower distributions (0.274–0.297 ± 0.01) (Figure 3D).

#### 2.1.2. Cargo Protection Under Physiological Stress

The ability of the nanocomplexes to protect genetic cargo from enzymatic and environmental degradation was evaluated under physiologically relevant conditions, as summarized in Figure 4. Naked pDNA exposed to 10% fetal bovine serum (FBS), trypsin, and culture medium (Lanes 3–5) exhibited a migration pattern and band intensity comparable to those of the untreated control (Lane 2). Complexed pDNA exhibited minimal band migration, indicating protection within the complex (Lanes 11–13 and 15–17). Integrity was further confirmed by treatment with 10% sodium dodecyl sulfate (SDS); the competitive displacement of pDNA by the anionic surfactant successfully released intact supercoiled plasmid (Lanes 14 and 18), validating that the genetic cargo remained structurally sound and accessible upon complex dissociation. Notably, an artefact was observed in the SDS lanes, where the anionic surfactant was present above its critical micelle concentration and spontaneously self-assembled into negatively charged supramolecular aggregates that migrated through the TAE-agarose matrix. The non-specific partitioning of the lipophilic GreenSafe nucleic acid stain into the hydrophobic cores of the migrating detergent micelles led to the encapsulation of GreenSafe, which emitted a false-positive fluorescent signal upon UV excitation, appearing as a smear at a molecular weight distinct from that of pDNA.

#### 2.1.3. In Vitro Hemocompatibility and Biocompatibility

To ensure suitability for systemic or local administration, hemocompatibility was assessed using rat red blood cells (RBCs). Both nanocomplexes prepared at N/P ratios of 0.5 and 1 induced hemolysis rates of 2.2% and 4.8%, respectively, below the accepted 5% hemolysis threshold for biomaterials (Figure 5A,B). Furthermore, cytotoxicity assays performed in HA1800 human astrocytes demonstrated high biocompatibility, with cell viability exceeding 90% 72 h post-transfection (Figure 5C). These results indicate that the TMZ-WRAP5/T7/pDNA system is non-cytotoxic to normal glial cells.

### 2.2. U87 MG 2D In Vitro Studies

#### 2.2.1. pDNA Cellular Internalization

Confocal fluorescence microscopy of fixed cells was used to visualize the cellular uptake of the developed peptide/pDNA complexes at N/P 1 in U87 MG cells 4 h after transfection (Figure 6). The nuclei were stained with Hoechst 33342 (blue), and pDNA was labeled with FITC (green). Non-transfected control cells exhibited no detectable FITC signal, validating the absence of background autofluorescence and the specificity of the pDNA labeling (Figure 6A). The targeted TMZ-WRAP5/T7/pDNA complexes (Figure 6C) showed significantly higher intracellular fluorescence compared with non-targeted analogs (Figure 6B). Quantification of Mean Fluorescence Intensity (MFI) confirmed a 2.5-fold increase in internalization for the T7-functionalized complexes: 15 arbitrary units (AU) vs. 6 AU (Figure 6D).

#### 2.2.2. p53-Mediated Expression, Apoptosis, and Cellular Viability

To evaluate the functional success of p53 gene delivery and expression, protein levels were quantified 24 h after transfection via ELISA. The formulated peptide nanocomplexes successfully mediated gene delivery and p53 expression, as shown in Figure 7. The TMZ-WRAP5/T7/pDNA system achieved the highest p53 expression levels (**** *p* ≤ 0.0001), significantly outperforming all other groups, including non-targeted and naked pDNA controls (Figure 7A). To further elucidate the molecular mechanisms triggered by this upregulation, Bax expression and activity of the intrinsic apoptotic pathway were subsequently evaluated. Consistent with the p53 results, all peptide complexes increased Bax expression in U87 MG glioma cells; however, the TMZ-WRAP5/T7/pDNA system at N/P 1 produced the greatest increase (Figure S4). Interestingly, no significant difference was observed between N/P ratios of 0.5 and 1 in this system, suggesting similar Bax induction efficiency at these concentrations. This pro-apoptotic signaling was further corroborated by a significant increase in the activities of both initiator caspase-9 and executioner caspase-3. Notably, the targeted system at N/P 1 induced the most robust activation of both caspases (*** *p* ≤ 0.0001) compared with the other peptide/pDNA nanocomplexes (Figure 7B,C). The therapeutic impact of these nanocomplexes was further assessed by measuring cell viability 72 h post-transfection. Treatment with TMZ-WRAP5/T7/pDNA at N/P 1 resulted in a statistically significant 27% reduction in viability compared with untreated controls (*** *p* ≤ 0.0001; Figure 7D). Although modest, this reduction is classified as weak to moderate according to standard cytotoxicity classification scales; it is important to note that the MTT assay primarily reflects mitochondrial metabolic activity and may not fully capture the cumulative effect of p53-mediated growth arrest and programmed cell death in resistant GB models.

### 2.3. Optimization of 3D Glioblastoma Spheroid Model

#### 2.3.1. Impact of Serum Concentration in Spheroid Formation

The influence of FBS concentration on U87 MG GB spheroid formation was investigated using the LOT in agarose-coated wells, as summarized in Figure 8 [55]. Under serum-free conditions (0% FBS), the cells failed to aggregate into cohesive structures, resulting in dispersed clusters and significant cell loss during medium exchange. In contrast, supplementation with 5% or 10% FBS supported the formation of compact, spherical aggregates by day 3 (Figure 8A). Because no statistically significant differences in diameter, area, or circularity were observed between 5% and 10 % FBS (ns, *p* > 0.05), the 5% concentration was selected for subsequent studies to minimize potential interference with transfection efficiency (Figure 8B–D).

#### 2.3.2. Design of Experiments for Model Optimization

##### Experimental Design

A central composite face-centered (CCF) design was implemented to systematically optimize the culture parameters for U87 MG GB spheroid formation. Three numerical factors were used as input variables: agarose coating solution volume/well (50–100 µL); agarose concentration (1–3 % *w*/*v*); and initial cell seeding density (500–10,000 cells/well). The experimental matrix comprised 17 runs, including three replicates at the center point (Runs 5, 7, and 15) to assess model reproducibility and pure error. Spheroid assembly was successful across all tested conditions, with measured diameters ranging from 359 µm to 523 µm and areas ranging from 82,108 µm^2^ to 185,933 µm^2^ (Table S3).

##### Model Fitting

Response surface models were fitted to the experimental results using Design-Expert software. For both diameter and area, a quadratic polynomial model was selected as the highest-order polynomial model. Statistical evaluation confirmed the robustness of these models, as presented in Table 2. The Predicted R^2^ values were in close agreement with the Adjusted R^2^ values, with differences below the 0.2 threshold, indicating high predictive power. Furthermore, the Adequate Precision ratios, which measure the signal-to-noise ratio, were 47.81 for diameter and 15.70 for area. Both values significantly exceeded the recommended limit of 4, confirming that the models could be used to navigate the design space.

The reliability of the models was further visually verified through predicted-versus-actual response plots (Figure 9). The tight distribution of data points along the 45-degree diagonal confirmed that the regression equations accurately capture the correlations between the independent variables and the physical outcomes.

##### Analysis of Variance and Factor Interactions

ANOVA was performed to determine the statistical significance of the model terms (Table S4). The high F-values (270.53 for diameter; 21.79 for area) and associated *p*-values (*p* < 0.0001 and 0.0003, respectively) indicated that both models are statistically significant. Notably, the lack-of-fit tests were non-significant for both responses (*p* > 0.05), suggesting that the models adequately represent the experimental data without excluding critical terms. Among the factors studied, cell seeding density (Factor C) emerged as the dominant contributor to variability in spheroid size. For diameter, significant two-factor interactions were observed between agarose volume and concentration (AB), and between volume and cell number (AC). Additionally, the significance of the quadratic term for cell number (C^2^) in both responses reveals a nonlinear relationship.

##### Response Optimization and Validation

To identify the optimal culture conditions, numerical targets were set to achieve a spheroid diameter of 400 µm and an area of 100,000 µm^2^. Optimization based on desirability scores (ranging from 0.938 to 1.000) yielded 88 potential solutions; the top 20 solutions are available in the SM (Table S5). The top-ranked condition, consisting of 97.5 µL of 2.8% agarose and a seeding density of 5441 cells/well, was selected for experimental validation. Validation experiments were conducted in quadruplicate (n = 4) to assess the model’s predictive accuracy (Table 3). The experimentally determined mean diameter (404.96 ± 4.51 µm) deviated by only 1.24% from the 400 µm target and fell precisely within the 95% prediction interval (PI). While the mean area (115,396 ± 8514 µm^2^) showed a higher deviation (15.4%) from the nominal target, it remained within the 95% PI.

### 2.4. Nanocomplex-Induced Growth Suppression and Structural Disintegration in 3D Glioblastoma Spheroids

Following the optimization of the 3D model, U87 MG spheroids (~400 µm diameter) were treated on day 3 after formation (D0) with a single dose of TMZ-WRAP5/T7/pDNA nanocomplexes (N/P 1) at increasing concentrations (5–50 µg/mL pDNA), while untreated spheroids served as the control group. Spheroid morphology and growth were monitored from day 0 to day 5 post-treatment. Visual monitoring revealed progressive disintegration and the accumulation of detached cells surrounding the treated spheroids, a phenomenon not observed in controls (Figure 10). This debris, consisting of peripheral cells shed from the spheroid, suggests progressive disintegration of the 3D tumor architecture upon treatment.

The nanocomplex exhibited a dose-dependent inhibition of spheroid growth (Figure 11). At lower concentrations, growth inhibition increased significantly with increasing dose. However, these spheroids reached a plateau and entered a regrowth phase. In contrast, the highest dose (50 µg/mL pDNA) achieved maximal growth suppression by day 3 post-transfection, resulting in a 41% decrease in diameter and a 46% decrease in area relative to the untreated control at that corresponding time point (****, *p* ≤ 0.0001). Although a plateau was observed following the initial inhibitory phase, the regrowth shown at lower doses was not observed at 50 µg/mL during the study period. This substantial reduction in spheroid size confirms the therapeutic potential of the targeted co-delivery system.

### 2.5. Nanocomplexes Penetration and Internalization into Spheroids

To evaluate the ability of the targeted nanocomplexes to overcome the physical barriers within the 3D tumor microenvironment, the penetration depth of FITC-labeled nanocomplexes was monitored in U87 MG spheroids using confocal fluorescence microscopy (Figure 12). Qualitative analysis of the z-stack slices revealed that while non-targeted complexes (TMZ-WRAP5/pDNA) were primarily localized in the peripheral cell layers, the T7-functionalized complexes (TMZ-WRAP5/T7/pDNA) achieved deeper penetration, with green fluorescence detectable throughout the entire spheroid (Figure 12A). This observation was quantitatively corroborated by radial profile analysis, displaying the normalized integrated intensity as a function of the spheroid radius (Figure 12B). The targeted system exhibited substantially higher fluorescence levels than the non-targeted analog and untreated controls. Specifically, at a radius of 100 µm (corresponding to the mid-region of the spheroid), the targeted system exhibited a significant increase in uptake, demonstrating the important role of T7-mediated interactions in facilitating deep tissue penetration within the 3D glioma model (Figure 12C).

### 2.6. Assessment of Cytotoxicity via Live/Dead Spheroid Assay

The therapeutic efficacy of the co-delivery system was further assessed using a Calcein-AM/propidium iodide (PI) live/dead assay 72 h post-transfection (Figure 13). Representative images of all experimental conditions are provided in the SM (Figure S5). Spheroids treated with the targeted TMZ-WRAP5/T7/pDNA complexes exhibited a marked increase in the PI signal (red) compared with the untreated control (Figure 13A). Quantitative analysis of the corrected total cell fluorescence (CTCF) for the PI channel revealed that the targeted system induced the highest level of cell death (*** *p* ≤ 0.001), outperforming all other experimental groups, while being comparable to the non-targeted system (Figure 13B). These results demonstrate that the co-delivery of TMZ and pDNA not only suppresses growth but also induces widespread cell death throughout the 3D GB spheroid.

## 3. Discussion

To advance the clinical translation of GB therapies, the present study describes the development of a targeted peptide-based nanosystem for TMZ/p53 co-delivery to enhance tumor targeting and intracellular delivery while overcoming resistance in GB through TfR-mediated cellular uptake. Furthermore, the study involved optimizing a reproducible spheroid model, which more accurately mimics the GB microenvironment than conventional 2D cultures, to confirm the therapeutic potential of this dual-action therapeutic system. The self-assembly of the TMZ-WRAP5/T7/pDNA nanocomplexes is driven by electrostatically co-precipitation. This process is mediated by electrostatic attraction between the cationic amine groups (N) of the peptides and the anionic phosphate groups (P) of the pDNA, resulting in a stable, non-covalent nanostructure. Our findings identified the N/P ratio as the primary determinant of the system’s physicochemical profile. While the T7 targeting peptide mediates partial pDNA charge neutralization, its charge density is insufficient to achieve complete condensation. Consequently, unused anionic sites remain available on the pDNA for subsequent interaction with the more highly charged WRAP5 peptide. Electrophoretic mobility assays confirmed that WRAP5 acts as the effective cationic driver, facilitating complete pDNA sequestration and the formation of compact nanostructures at higher N/P ratios.

Notably, the high TMZ incorporation efficiency (86.6–88.7%) was largely independent of the N/P ratio. These findings suggest that the drug-loading capacity is primarily determined by the initial peptide-drug conjugation efficiency rather than the subsequent pDNA condensation stage. While spectrophotometric analysis provided strong baseline data, future studies utilizing high-performance liquid chromatography (HPLC), for example, may provide a more detailed assessment of drug retention within the peptide matrix.

The SEM analysis revealed well-dispersed spherical nanostructures, a morphology that is advantageous for clinical translation. Although recent literature has explored the benefits of non-spherical nanoparticles, such as prolonged circulation times, spherical nanoparticles remain widely recognized as a preferred nanoparticle morphology for their efficient cellular internalization. Their lower membrane-bending energy requirements during endocytosis facilitate more efficient membrane wrapping and uptake compared with high-aspect-ratio structures [56,57,58,59].

The progressive reduction in hydrodynamic diameter and the charge inversion observed at higher N/P ratios indicated strong electrostatic interactions, leading to enhanced pDNA condensation and the formation of more compact nanostructures [60,61]. A positive surface charge enhances non-specific interactions with the negatively charged cell membranes, thereby facilitating cellular internalization; however, it also increases cytotoxicity toward healthy cells [62]. This limitation was addressed by incorporating the T7 ligand, which targets the overexpression of TfR on glioma cells to improve tumor specificity [63]. The slightly larger size of the TMZ-WRAP5/T7/pDNA complexes may be attributed to the higher payload capacity, in agreement with previous literature studies [64,65], yet the system remains close to the 200 nm range of diameter commonly considered optimal for targeted therapies [66].

A significant challenge in non-viral gene delivery is balancing the extracellular stability with efficient intracellular release [61,67,68]. The maintenance of pDNA integrity under physiological stress (FBS, trypsin, and culture medium), and its subsequent release in its supercoiled conformation upon SDS-mediated displacement, confirms that the nanocomplex functions as a stimuli-responsive shield.

Biological safety was evaluated through hemocompatibility and viability assays in human astrocytes. The hemolysis rate (<5%) and high viability (>90%) in HA1800 astrocytes suggest a favorable safety window. Hemolysis serves as a critical safety parameter for intravenous formulations, as membrane disruption of erythrocytes can lead to hemolytic anemia and other complications [69,70,71]. Despite the small increase in the hemolysis rate at N/P 1 (4.8%), possibly related to the higher positively charged surface of the complexes at this ratio, the values remain within the ISO 10993-4 and ASTM standards for non-hemolytic biomaterials [72,73]. Furthermore, by focusing on astrocytes, the most abundant glial cells in the central nervous system, we were able to assess potential off-target toxicity and mimic the healthy tissue context surrounding GB tumors [74,75,76]. The high astrocyte viability after nanocomplex transfection indicated that the system avoids off-target toxicity.

Using fluorescence confocal microscopy, it was possible to confirm the cellular uptake of the developed nanocomplexes. The 2.5-fold increase in cellular uptake mediated by T7 functionalization underscores the efficacy of active targeting. By exploiting the overexpression of TfR on GB cells, a consequence of the high iron metabolic demands of malignant proliferation, the TMZ-WRAP5/T7/pDNA system achieves superior internalization compared with non-targeted analogs [77,78,79]. Functional efficacy was further validated by the upregulation of p53 protein levels and the concomitant activation of the apoptotic cascade. The strength of this system lies in its dual-pronged strategy: TMZ induces DNA damage, while the delivered *TP53* gene, through p53 supplementation, restores apoptotic pathways that are frequently disrupted in high-grade gliomas. Our expanded molecular analysis confirms that this occurs via the intrinsic mitochondrial pathway. The upregulation of the pro-apoptotic protein Bax and the subsequent activation of initiator caspase-9 and executioner caspase-3 provide a mechanistic map of the treatment’s effect. Interestingly, the lack of a significant difference in Bax expression between N/P 0.5 and 1 suggests that even lower peptide concentrations are sufficient to trigger the initial stages of mitochondrial outer membrane permeabilization [80], though the higher N/P ratio ultimately yielded more robust downstream caspase activation and cargo protection.

This co-delivery approach resulted in a significant reduction in U87 MG cell viability (27%). However, we acknowledge the limitations of the MTT assay, including that it provides an indirect estimate of cell viability, presents limited sensitivity, and has high variability. Multiple factors, such as cell line, seeding density, incubation time, culture medium composition, and MTT concentration, influence the MTT readouts [81,82,83,84]. Therefore, these results should be viewed as an indicator of therapeutic potential rather than a definitive measure of cytotoxicity. More sensitive and informative approaches, such as flow cytometry-based assays, would provide a more comprehensive and reliable evaluation of the therapeutic potential of TMZ-WRAP5/T7/pDNA nanocomplexes.

To overcome the limitations of 2D monolayers and the variability of existing 3D GB spheroid protocols [43], a U87 MG spheroid model has been optimized by DoE. Our findings emphasize serum concentration as a critical modulator of spheroid architecture; 5% FBS proved sufficient for maintaining structural integrity, with no added benefit at 10%. Remarkably, the literature does not fully agree regarding serum requirements for GB spheroids. While some protocols employ serum-free conditions to enrich cancer stem-like cell populations [85,86,87], others rely on serum-supplemented media to promote spheroid integrity and growth, with serum-free conditions resulting in spheroid shrinkage, reduced density, and increased cell detachment [42,43,88,89]. This variability likely reflects the heterogeneity of GB, where the behavior and responses to stimuli vary significantly across different cell lines [89,90].

The transition from a traditional OFAT approach to a CCF design was critical. The DoE revealed that cell density is the dominant factor influencing spheroid formation, while also uncovering significant interactions between agarose volume and concentration. This non-linear relationship highlights the complexity of 3D cell–cell aggregation and justifies the use of response surface modeling to ensure reproducibility. The resulting protocol of the optimization process (93.5 µL of 2.808% agarose, 5441 cells/well) consistently yielded uniform spheroids on day 3, across multiple independent experiments, regardless of cell passage number—a parameter known to influence cell behavior, proliferation rates, and spheroid growth [91,92,93].

In the 3D spheroid model, the TMZ-WRAP5/T7/pDNA nanocomplexes demonstrated potent, dose-dependent growth inhibition. The observed desegregation of cells from the spheroid periphery suggests effective targeting of the proliferative outer zone, a region typically more susceptible to damage from delivery systems [94,95,96]. The growth stagnation and partial regrowth observed after day 3 are common consequences of single-dose treatments and likely reflect the exhaustion of the therapeutic payload or the selection of resistant cell subpopulations.

Meanwhile, penetration studies provide a physical explanation for the observed therapeutic success in growth inhibition. The 3D microenvironment presents a challenging barrier to nanoparticle diffusion due to high interstitial pressure and dense extracellular matrix [97]. However, the T7-functionalized complexes achieved significantly higher radial intensity compared with non-targeted analogs. This suggests that T7-mediated targeting of the TfR does not merely enhance cellular uptake in monolayer U87 MG cells but may also facilitate transport through the cell layers, allowing the complexes to reach the inner spheroid. The marked increase in PI fluorescence across the treated spheroids further indicates that the observed reduction in spheroid growth is primarily attributable to treatment-induced cell death rather than simple growth arrest. While the non-targeted system showed activity comparable to the targeted system in the Live/Dead assay, future studies with larger sample sizes and quantitative apoptosis analysis will be important to further resolve these differences. Additionally, the inclusion of nanocomplexes formulated with unrelated or mutant p53 plasmids as negative controls in future studies would further elucidate the specific biological impact of the therapeutic cargo.

To enhance the translational potential of the developed nanoplatform, several aspects merit further investigation. Future studies should evaluate multiple-dose regimens to better mimic clinical therapeutic schedules. Incorporating spatial imaging would clarify nanocomplex penetration depth, particularly into the hypoxic core. In addition, viability assays, apoptosis markers, and proliferation indices would provide a deeper mechanistic insight into treatment response and resistance in the 3D context.

While the T7 ligand was selected to exploit TfR overexpression for potential brain targeting, our current study primarily demonstrates enhanced uptake and efficacy in 2D and 3D tumor models. These results suggest a putative BBB transport capability based on the characteristics of the T7 peptide; however, direct translocation across the BBB was not experimentally verified in this work. The absence of a specific BBB model, such as an in vitro Transwell system or an in vivo biodistribution study, represents a limitation that will be addressed in future evaluations. Furthermore, direct analysis of the specificity of the functionalization, for example, through knock-out models or competition assays, is required to fully characterize the TfR-mediated pathway.

Furthermore, while our system demonstrates high efficacy in the implemented models and potential as a candidate strategy, it is valuable to compare it with other emerging biological strategies. For instance, unlike targeted toxin-based bispecifics, which may face challenges related to drug toxicities, immunogenicity issues, perceived limitations in bulk drug production and formulation, and the often-transient nature of clinical responses observed in clinical trials, our peptide-based nanocarrier provides a stable vehicle for prolonged combined action of gene and drug. However, bispecifics may offer superior specificity, for example, in vascular targeting, a facet we aim to explore in future functionalization studies [98,99]. Also, while lipid-based nanoparticles have emerged as the most promising drug delivery system for the treatment of GB, peptide-based systems such as TMZ-WRAP5/T7/pDNA may offer advantages in modular functionalization and intrinsic cell-penetrating capacity [100,101].

Additionally, while we established safety for intravenous routes, alternative strategies, such as intranasal delivery and intracranial injection, could further improve brain bioavailability by bypassing the BBB. The intracranial delivery route is particularly well-suited for targeting residual tumorigenic cells in the resection cavity. By applying the TMZ-WRAP5/T7/pDNA complexes as a localized “preventive” option, embedded for example in a hydrogel, immediately after surgery, we may (as a hypothesis) effectively eliminate infiltrative cells that typically drive recurrence and metastatic-like spread within the brain parenchyma, potentially extending the disease-free interval [102].

Ultimately, the demonstrated dual-action capability, deep penetration into 3D tumor models, and validated efficacy of this targeted nanosystem represent a promising paradigm shift in GB treatment, providing a potent multifunctional strategy to bypass biological resistance and advance toward more precise, clinically viable therapies.

## 4. Materials and Methods

### 4.1. Materials

The WRAP5 peptide (NH_2_-LLRLLRWWWRLLRLL-CONH_2_) was synthesized via Fmoc/tert-butyl (tBu) solid-phase peptide synthesis using the LibertyBlue™ Microwave Peptide Synthesizer (CEM Corporation, Matthews, NC, USA) equipped with a Discover™ module (CEM Corporation, NC, USA) operating at 2450 MHz, as previously described [61]. The T7 peptide (Ac-HAIYPRH-NH_2_) was a gift from Synpeptide Co., Ltd. (Pukou, Nanjing, China). Temozolomide (TMZ) was sourced from Frilabo (Lisbon, Portugal).

The (GFP)-p53 plasmid (5894 bp) was purchased from Addgene (Cambridge, MA, USA), amplified in *Escherichia coli* DH5α cells, and purified according to previously described protocols [61]. 3-(4,5-dimethylthiazol-2-yl)-2,5-diphenyltetrazolium bromide (MTT) and Fluorescein isothiocyanate isomer I (FITC) were purchased from Sigma-Aldrich (St Louis, MO, USA). Hoechst 33342, Dimethyl sulfoxide (DMSO), and TritonX-100 were supplied by Thermo Fisher Scientific (Waltham, MA, USA). SDS was obtained from HiMedia Laboratories GmbH (Maharashtra, India). Agarose and GreenSafe Premium were obtained from NZYTech (Lisbon, Portugal). Dulbecco’s Modified Eagle’s Medium (DMEM) with high glucose and stable L-glutamine was sourced from Biowest (Nuaillé, France). A Milli-Q system from Millipore (Billerica, MA, USA) was used for water purification, and ultrapure-grade water was used for all solutions.

Human malignant GB cell line, U87 MG, was kindly provided by Dr. Joseph Costello (University of California, San Francisco, CA, USA). The human normal astrocyte cell line, HA1800, isolated from human brain (cerebral cortex) and cryopreserved, is from the American Type Culture Collection (ATCC, Manassas, VA, USA). Rat blood was obtained through the RISE-Health, UBI bioterium (animal facility) in accordance with institutional ethical guidelines.

### 4.2. Preparation of TMZ-Loaded Peptides

The TMZ-peptide mixture was prepared following a previously established protocol [61]. Briefly, TMZ-WRAP5 was prepared by mixing 150 µL of WRAP5 peptide solution (2 mg/mL) with 360 µL of TMZ in absolute ethanol solution (0.5 mg/mL) and dispersing in 8 mL of ultrapure water via 90 s of sonication. The mixture was frozen overnight and lyophilized for 24 h. Post-lyophilization, the sample was reconstituted in 200 µL of ultrapure water.

### 4.3. Formation of Peptide/pDNA Complexes

The peptide/pDNA complexes, TMZ-WRAP5/T7/pDNA (A), were formed by adding 50 µL of TMZ-WRAP5 solution dropwise to 150 µL of T7 and pDNA (1 µg) under vortexing for 1 min. Formulations were prepared in ultrapure water and equilibrated at room temperature for 25 min, then centrifuged at 13,500× *g* for 20 minutes at 4 °C. The resulting pellets were stored at 4 °C for immediate use or at −20 °C for longer periods before use.

A second approach was considered for the formulation of the complexes, WRAP5/T7/TMZ/pDNA (B); all corresponding data are presented in the SM.

To assess TMZ complexation efficiency, a standard curve was established via spectrophotometry at 330 nm using the NanoPhotometer™ ((Implen, Inc.,Westlake Village, CA, USA) ranging from 50 to 3200 µg/mL in ultrapure water as detailed in Figure S2 (SM).

The complexation efficiency of TMZ was determined using Equation (1):

(1) $$Drug\;load\;efficiency\;\left(\%\right)=\frac{ci-cs}{ci}\times 100$$

where *ci* and *cs* correspond to the initial drug concentration and drug concentration remaining in the supernatant, respectively.

### 4.4. Agarose Gel Electrophoresis

Electrophoresis was conducted for 30 min at 150 V in 1% (*w*/*v*) agarose gel, prepared in 1x TAE buffer (40 mM Tris base, 20 mM acetic acid, 1 mM EDTA at pH 8.0) and stained with GreenSafe. The gel visualization was performed using the Uvitec Fire-Reader system (Uvitec Limited, Cambridge, UK).

### 4.5. Scanning Electron Microscopy

The geometry and morphology of the complexes were characterized using SEM. After formulation, the recovered pellet was suspended in 40 µL of 2% tungsten aqueous solution. The sample was pipetted onto round-shaped coverslips and dried overnight at room temperature. An Emitech K550 (London, UK) sputter coater was used to coat the samples with gold. The complexes were visualized using a Hitachi S-2700 SEM (Tokyo, Japan) operating at 20 kV at various magnifications.

### 4.6. Particle Size and Zeta Potential Measurements

Average size, PdI, and zeta potential were characterized at 25 °C using a Zetasizer Nano ZS (Malvern Instruments, Malvern, UK) equipped with Malvern Zetasizer software v6.34. Following resuspension of the pellet in 5% glucose with 1 mM NaCl solution, particle size and PdI were determined by Dynamic Light Scattering (DLS) via non-invasive backscattering (NIBS) using a 633 nm He-Ne laser. Surface charge was measured through Electrophoretic Light Scattering (ELS) using an M3-PALS (Phase Analysis Light Scattering) laser.

### 4.7. Stability Assays

The stability of TMZ-WRAP5/T7/pDNA complexes (N/P ratios 0.5 and 1) was evaluated under simulated physiological conditions by incubating nanocomplex pellets with 10% FBS, trypsin, and DMEM high glucose with stable L-glutamine for 4 h at 37 °C. Following incubation, pDNA integrity and release were monitored via 1% agarose gel electrophoresis (150 V, 30 min). A naked pDNA sample (1 µg, non-complexed) exposed to identical conditions and incubated as previously described, and another that did not undergo incubation, served as controls. To assess the integrity of pDNA after decomplexation, 10% SDS was added to samples that had been incubated with culture medium and incubated for 10 min before electrophoresis analysis.

### 4.8. Hemolysis Test

The hemocompatibility of TMZ-WRAP5/T7/pDNA complexes was evaluated via a hemolysis assay using rat RBCs. Erythrocytes were isolated from heparinized blood (2 mL) by centrifugation (3000 rpm, 15 min, 4 °C) and washed with 0.85% (*w*/*v*) saline (NaCl) solution until the supernatant was clear. A 3–5% (*v*/*v*) RBC suspension was prepared in PBS (pH 7.4), and 900 µL aliquots were incubated with 100 µL of resuspended complexes for 1 h at 37 °C. PBS and 1% Triton X-100 served as negative and positive controls, respectively. Following incubation, samples were centrifuged (8000 rpm, 20 min), and the absorbance of the supernatant was measured at 576 nm using a UV-visible spectrophotometer (BioRad, Vienna, Austria). The percentage of hemolysis was determined using Equation (2):

(2) $$Hemolysis\;\left(\%\right)=\frac{{Abs}_{Sample}-{Abs}_{Negative\;Control}}{{Abs}_{Positive\;Control}-{Abs}_{Negative\;Control}}\times 100$$

### 4.9. 2D Cell Culture

#### 4.9.1. Monolayer 2D Culture Model

HA1800 and U87 MG cell lines were maintained in 25 cm^2^ T-flasks using DMEM high glucose with stable L-glutamine medium pH 7.45, supplemented with 10% heat-inactivated FBS and 1% (*v*/*v*) penicillin-streptomycin (100 µg/mL each). Cultures were kept at 37 °C in a humidified atmosphere (5% CO_2_) and sub-cultivated upon reaching confluence to ensure exponential growth.

#### 4.9.2. Cellular Viability in Astrocytes

Biocompatibility of the complexes was evaluated in HA1800 astrocytes using the MTT colorimetric assay. Briefly, astrocytes were seeded in 96-well plates at a density of 10,000 cells/well in DMEM high glucose with stable L-glutamine supplemented with 10% FBS and maintained at 37 °C under a 5% CO_2_ atmosphere. At 24, 48, and 72 h post-transfection, cells were incubated with MTT (5 mg/mL) for 2 h. The culture medium was subsequently replaced with 200 µL of DMSO, and the plates were agitated for 30 min in the dark to solubilize the formazan crystals. Absorbance was measured at 570 nm using a Benchmark Microplate Reader (BioRad, Vienna, Austria). Non-transfected and ethanol-treated cells served as positive and negative controls, respectively, and cell viability (%) was calculated according to Equation (3):

(3) $$Cellular\;viability\;\left(\%\right)=\frac{{\left[A\right]}_{test}}{{\left[A\right]}_{control}}\times 100$$

where [A]_test_ is the absorbance of the test sample and [A]_control_ is the absorbance of the positive control sample.

#### 4.9.3. FITC Plasmid Labeling

To visualize cellular uptake, pDNA (2 µg) was fluorescently labeled with 2 μL of FITC solution (in sterile anhydrous dimethyl sulfoxide, 50 mg/100 µL) and 63 μL of labelling buffer (0.1 M Sodium Tetraborate, pH 8.5). The reaction mixture was stirred for 4 h at 4 °C in the dark. To finalize the reaction, 2.5 volumes of ethanol, 100% (212.5 μL) and one volume of 3 M NaCl (85 μL) were added. Samples were incubated at −20 °C overnight. Following centrifugation (10,000× *g*, 30 min, 4 °C), the labeled pDNA pellet was washed with 75% ethanol to remove unbound dye before its utilization in nanocomplex preparation.

#### 4.9.4. Cellular Uptake/Internalization

The cellular uptake and intracellular distribution of FITC-labeled peptide/pDNA complexes were evaluated in U87 MG GB cells using a Zeiss LSM 710 confocal laser fluorescence microscopy (CLSM) (Carl Zeiss SMT, Inc., Oberkochen, Germany). Cells were seeded at a density of 100,000 cells/well in 12-well plates and transfected with complexes (1 µg pDNA-FITC/well) for 4 h. The cells were then fixed with 5% (*w*/*v*) paraformaldehyde (15 min) and the nucleus stained with Hoechst 33342 (1:2000, 10 min). Imaging was conducted using a 63× oil immersion objective and analyzed with the ZEN microscopy software 3.7. The images were acquired with the laser and filters corresponding to the respective fluorophores used, namely Hoechst (445/450 nm) and FITC (525/550 nm). Processed and analyzed with Zeiss Zen (blue edition) software version 3.14 (Carl Zeiss SMT, Inc., Oberkochen, Germany). Microscopy experiments were performed at the Fluorescence Microscopy Unit of the RISE-Health, UBI, integrated in the national infrastructure PPBI—Portuguese Platform of Bioimaging.

#### 4.9.5. p53 Quantification

Intracellular p53 levels in U87 MG cells were quantified 24 h post-transfection using a p53 Pan ELISA kit (Roche Applied Science, Penzberg, Germany) according to the manufacturer’s protocol. Briefly, cells were harvested, washed with PBS, and detached via trypsinization, followed by centrifugation (5000 rpm, 5 min) and lysis to obtain protein extracts suitable for ELISA quantification. Absorbance readings were acquired using Benchmark Microplate Reader (BioRad, Vienna, Austria) at 450 nm, and p53 concentrations were calculated from the kit standard curve.

#### 4.9.6. Caspase-3 and Caspase-9 Quantification

Caspase-3 enzymatic activity and caspase-9 levels were quantified using the ApoAlert™ Caspase-3 Colorimetric Assay and Caspase-Glow^®^ 9 Assay (Promega, Madison, WI, USA) following the manufacturer’s instructions. Transfected U87 MG cells were harvested as previously described for p53 analysis. For caspase-3, the resulting p-nitroaniline (pNA) release was measured spectrophotometrically at 405 nm using a microplate reader. Caspase-9 activity was evaluated by measuring the luminescent signal resulting from the cleavage of a luminogenic substrate. Staurosporine-treated cells (1 µM) served as a positive control for apoptosis induction.

#### 4.9.7. Bax Quantification

Bax protein levels were quantified using the Human BAX ELISA Kit (No-EH0669) (Wuhan Fine-Biotech, Wuhan, China). Cells were harvested, lysed, and protein extracts were analyzed according to the manufacturer’s protocol, with absorbance measured at 450 nm. Briefly, Bax present in the lysates was captured by the immobilized primary antibody and detected using a horseradish peroxidase (HRP)-conjugated secondary antibody.

#### 4.9.8. Cellular Viability in U87 MG

The cytotoxicity of the complexes and cellular viability in U87 MG GB cells were evaluated using the MTT colorimetric assay. Similarly to when in astrocytes, cells were seeded in 96-well plates (10,000 cells/well) and maintained in the same culture conditions. At 72 h post-transfection, cells were incubated with MTT (5 mg/mL) for 2 h. The resulting formazan crystals were solubilized in 200 µL of DMSO for 30 min while protected from light, and absorbance was measured at 570 nm using a Benchmark Microplate Reader (BioRad, Vienna, Austria). Non-transfected and ethanol-treated cells served as positive and negative controls, respectively, with percentages quantified according to Equation (3).

### 4.10. 3D Cell Culture

#### 4.10.1. Spheroid 3D Culture Model

U87 MG GB spheroids were generated using an agarose-coated liquid overlay technique to provide a non-adherent surface for cell aggregation. Briefly, sterile 96-well flat-bottom plates were coated with a sterile agarose solution prepared in ultrapure water and allowed to solidify for 15–20 min at room temperature, without the lid to prevent condensation. Following the addition of 100 µL of culture medium supplemented with FBS, U87 MG cells were seeded dropwise into the center of each well to promote the formation of a single, uniform spheroid. Cultures were maintained at 37 °C in a 5% CO_2_ humidified atmosphere, with 50% of the medium replenished every three days to ensure nutrient availability while preserving spheroid architecture.

To optimize U87 MG spheroid formation, an initial study was conducted to evaluate the influence of FBS supplementation at the concentrations of 0%, 5%, and 10% (*v*/*v*) in DMEM high glucose with stable L-glutamine medium. In this study, U87 MG spheroids were formed by seeding 7000 cells/well in 96-well flat-bottomed plates coated with 75 µL of 1% (*w*/*v*) agarose.

#### 4.10.2. Design of Experiments

A DoE approach utilizing a CCD was employed to optimize the U87 MG spheroid formation protocol, targeting ideal morphology and size by day 3 or 4 of formation. Three numeric factors were considered as inputs: the concentration of the agarose coating solution, the volume of agarose solution added to the well, and the seeding cell density. These inputs were examined at three levels (−1, 0, +1), with the range defined based on preliminary studies. A quadratic model was explored through 17 experimental runs, including 3 central points, and analyzed using Design-Expert software v.13. Response surface methodology was applied using a generalized second-order polynomial model to identify the optimal experimental conditions, Equation (4):

(4) $$Y=\beta_{0}+\beta_{1}X_{1}+\beta_{2}X_{2}+\beta_{3}X_{3}+\beta_{11}X_{1}^{2}+\beta_{22}X_{2}^{2}+\beta_{33}X_{3}^{2}+\beta_{12}X_{1}X_{2}+\beta_{13}X_{1}X_{3}+\beta_{23}X_{2}X_{3}$$

where Y is the measured response, β’s are parameter coefficients, X’s are the input variables. Following statistical validation, the identified optimal point was adopted for all subsequent in vitro 3D studies.

#### 4.10.3. Complexes Transfection

TMZ-WRAP5/T7/pDNA complexes at N/P ratio of 1 (5/10/25/50 µg pDNA/mL) were transfected by day 3 post-seeding, considered day 0 (D0). Increasing doses were achieved by adding progressively higher amounts of nanoparticles per well, without altering the nanosystem composition. Images were acquired at D0 (pre-transfection) and after 1, 2, 3, 4, and 5 days of transfection (D1–D5), using a CKX41 Olympus microscope equipped with a 6.0 megapixels SP-500UZ Olympus camera (10× objective lens) (Olympus Deutschland GmbH, Hamburgo, Germany). Quantitative analysis of spheroid diameter, perimeter, and area was carried out via semi-automatic processing using the ImageJ software (version 1.54; NIH, Bethesda, MD, USA). Spheroid circularity was determined according to the Equation (5):

(5) $$Circularity=\frac{4\pi A}{P^{2}}$$

where A represents the area and P the perimeter of the spheroid.

#### 4.10.4. Penetration and Uptake into GB Spheroids

Spheroids were transfected on day 3 post-seeding with FITC-labeled TMZ-WRAP5/pDNA or TMZ-WRAP5/T7/pDNA complexes at an N/P ratio of 1 (50 µg/mL pDNA). After 24 h of incubation, spheroids were recovered and washed with PBS. Nuclei were stained with Hoechst 33342 (1:2000) for 1 h, followed by fixation with 5% (*w*/*v*) paraformaldehyde for 20 min. Spheroids were stored at 4 °C in PBS. For imaging, spheroids were transferred to Ibidi 8-well plates and visualized via CLSM using a 20× objective. Images were processed with Zeiss Zen software. For data analysis, quantification of Normalized Integrated Intensity by the Radius of the spheroid was done via a radial profile of the central z-stack slice, in FIJI software v.1.54t.

#### 4.10.5. Live/Dead Fluorescence Microscopy

Spheroid viability was assessed using the Calcein-AM/Propidium Iodide assay. On day 3 post-seeding, spheroids were transfected under various conditions (Naked pDNA, TMZ, TMZ-WRAP5, WRAP5/pDNA, TMZ-WRAP5/pDNA, and TMZ-WRAP5/T7/pDNA), at a corresponding concentration of the nanocomplex of 50 µg pDNA/mL, and incubated for 72 h. Spheroids were washed with PBS and stained with Calcein-AM (4 µM) and PI (5 µg/mL) for 45 min. After washing, nuclei were counterstained with Hoechst 33342 (1:2000) for 20 min. Spheroids were transferred to Ibidi plates for imaging using a 10× objective on a Zeiss LSM 710. Visual representation of the channels and merged image considers the SUM of all slices of the z-stack, and the z-stack representation was obtained through Zen software 3.7. Quantification of fluorescence was performed by considering the SUM of each channel, using the blue channel to determine the Region of Interest (ROI) and measuring the PI (red) channel signal. The corrected total cell fluorescence (CTCF) was calculated according to Equation (6):

(6) CTCF=Integrated Density−(Area ×Mean Fluorescence of Background)

The CTCF values were normalized to the spheroid area.

### 4.11. Statistical Analysis

Statistical analysis was conducted using one-way ANOVA and two-way ANOVA, followed by the Bonferroni post hoc test. All quantitative data are expressed as the mean ± SD. Differences were considered statistically significant when *p* < 0.05. Additionally: *, indicates *p* ≤ 0.05; ** indicates *p* ≤ 0.01; *** indicates *p* ≤ 0.001; **** indicates *p* ≤ 0.0001. Data analysis was carried out with GraphPad Prism v.10.0.0 software.

## 5. Conclusions

This study successfully established a targeted, peptide-based nanosystem for the co-delivery of TMZ and a p53-encoding pDNA to enhance therapeutic outcomes in GB. The developed nanocomplexes exhibited favorable physicochemical properties, high drug-loading efficiency, and a safe nanoparticle profile, as evidenced by hemocompatibility and healthy astrocyte assays. The incorporation of the T7 targeting peptide significantly enhanced cellular uptake and functional p53 expression, thereby reducing tumor cell viability. Critically, our results reveal that this effect is driven by activation of the intrinsic apoptotic pathway, characterized by upregulation of Bax and the sequential induction of the initiator caspase-9 and the executioner caspase-3.

Furthermore, by utilizing a DoE approach, we optimized a reproducible U87 MG spheroid model that more accurately mimics the structural barriers of the GB microenvironment than conventional 2D systems. In this 3D context, dose-dependent growth inhibition confirms the system’s therapeutic potential, supported by the superior radial penetration of the targeted complexes and direct visualization of cell death via PI staining. Overall, this work provides a comprehensive and adaptable framework for addressing key challenges in GB therapy, including tumor specificity and therapeutic resistance, representing a promising candidate for further preclinical development.

## Figures and Tables

**Figure 1:**
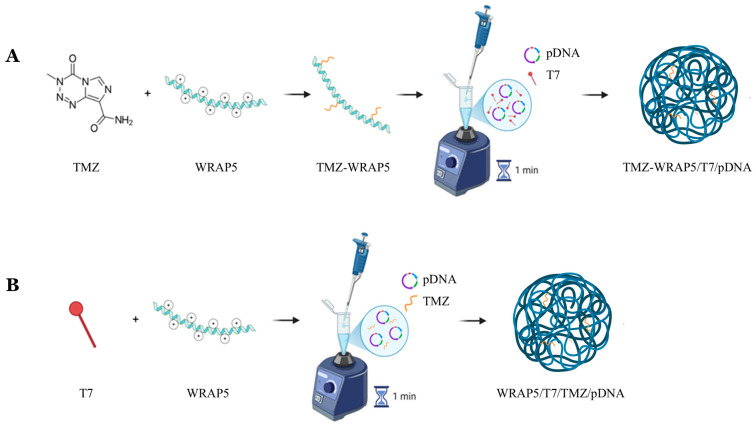
Schematic representation of TMZ-WRAP5/T7/pDNA (**A**) and WRAP5/T7/TMZ/pDNA (**B**) complex formation using a co-precipitation technique (dropwise for 1 min under vortexing). pDNA—Plasmid DNA; T7—HAIYPRH Peptide; TMZ—Temozolomide. Created with https://biorender.com (accessed on 2 May 2026).

**Figure 2:**
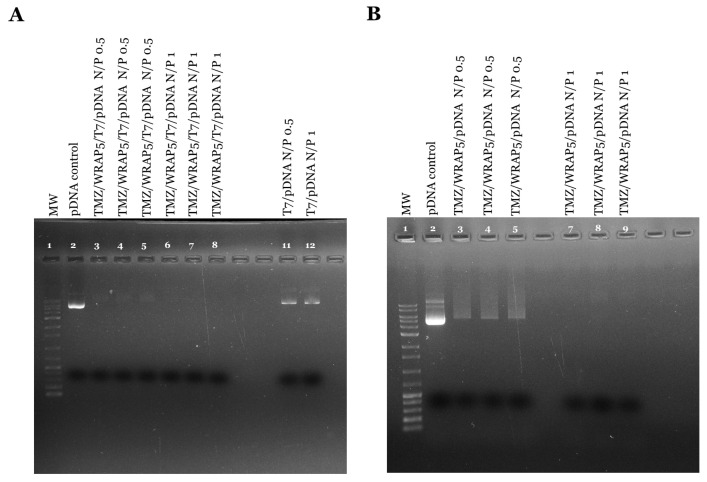
Electrophoretic analysis of peptide/pDNA complexes at different N/P ratios. Agarose gel electrophoresis was performed to assess the complexation behavior and stability of pDNA in complexes formulated at N/P ratios of 0.5 and 1. After formulation and centrifugation, the supernatants were loaded on the gel for analysis. (**A**) Lane 1: DNA molecular weight marker (MW); Lane 2: non-complexed pDNA; Lanes 3–5: TMZ-WRAP5/T7/pDNA at N/P 0.5; Lanes 6–8: TMZ- WRAP5/T7/pDNA at N/P 1; Lanes 11 and 12: T7/pDNA control complexes. (**B**) Lane 1: DNA molecular weight marker (MW); Lane 2: non-complexed pDNA; Lanes 3–5: TMZ-WRAP5/pDNA at N/P 0.5; Lanes 7–9: TMZ-WRAP5/pDNA at N/P 1.

**Figure 3:**
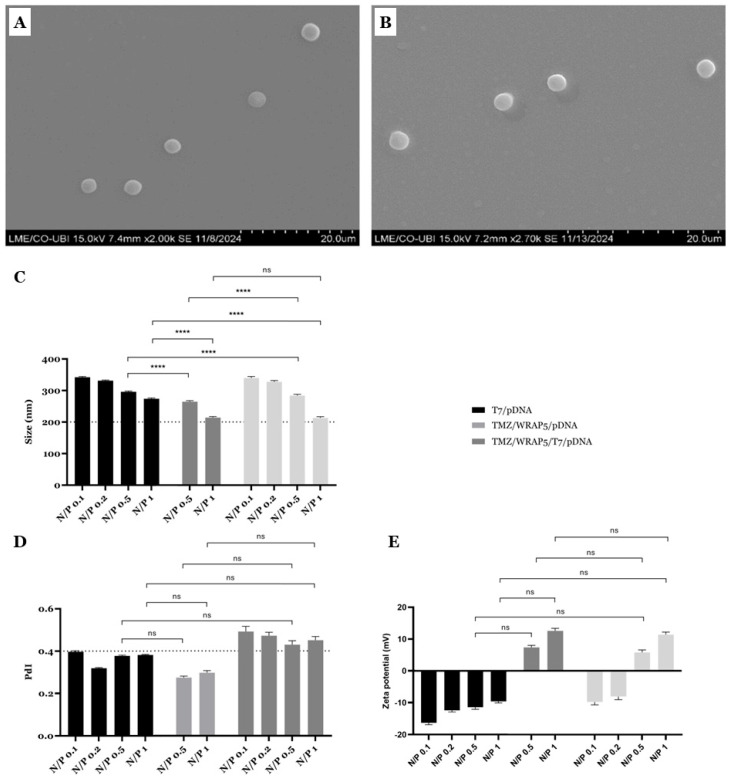
Morphology and physicochemical properties. Scanning electron micrographs of TMZ-WRAP5/T7/pDNA (**A**) and TMZ-WRAP5/pDNA (**B**) complexes at N/P ratio 1. Scale bar = 20 µm. Physicochemical properties of the different complexes at various N/P ratios. Mean size (**C**), PdI (**D**), and zeta potential (**E**). The values were calculated using data obtained from three independent measurements (mean ± SD, n = 3). Statistical analysis was performed using two-way ANOVA, followed by the Bonferroni post hoc test. The dotted lines in (**C**,**D**) represent the limits accepted for small particle size (<200 nm) and narrow size distribution (PdI < 0.4), respectively. **** *p* ≤ 0.0001; ns—statistically non-significant (*p* > 0.05).

**Figure 4:**
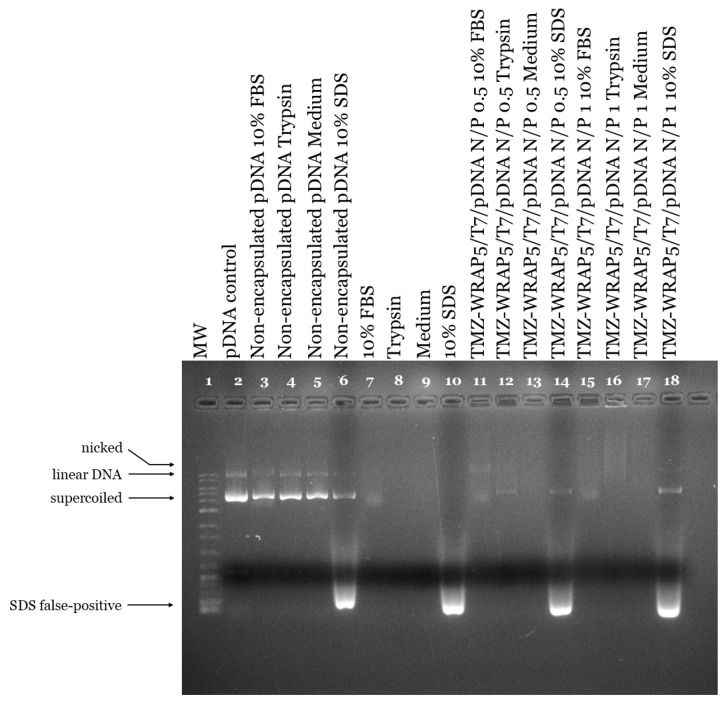
Electrophoretic analysis of pDNA protection capacity exhibited by TMZ-WRAP5/T7/pDNA complexes at N/P 0.5 and 1 after a 4 h incubation at 37 °C under four different conditions: 10% FBS, trypsin, cell culture medium (DMEM High Glucose w/stable Glutamine), and 10% SDS. Lane 1: DNA molecular weight marker (MW); Lane 2: non-complexed pDNA; Lanes 3–6: non-complexed pDNA in the respective incubation conditions; Lanes 7–10: negative controls; Lanes 11–14: TMZ-WRAP5/T7/pDNA N/P 0.5; Lanes 15–18: TMZ-WRAP5/T7/pDNA N/P 1.

**Figure 5:**
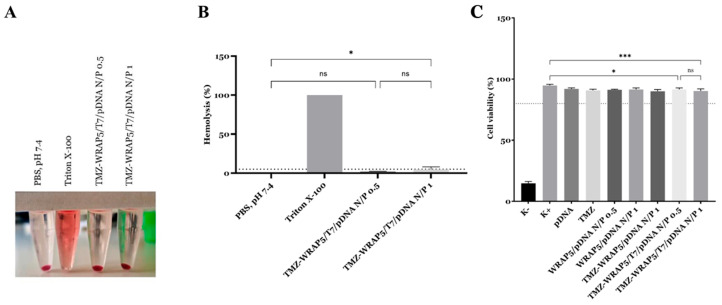
In vitro hemocompatibility and biocompatibility. Hemolysis test using rat RBCs treated with TMZ-WRAP5/T7/pDNA complexes developed at N/P ratios of 0.5 and 1 (**A**). Percentage of hemolysis (**B**). Cellular viability 72 h after transfection with peptide/pDNA complexes. HA1800 astrocytes transfected with different peptide/pDNA complexes at N/P ratios of 0.5 and 1 (**C**). Cells treated with ethanol served as the negative control (K−), non-transfected cells as a positive control (K+), and cells treated with naked pDNA and TMZ drug were used as controls. Statistical analysis: (**B**) data are presented as mean ± SD (n = 3), statistical analysis using one-way ANOVA followed by the Bonferroni post hoc test. (**C**) mean ± SD (n = 4), two-way ANOVA followed by the Bonferroni post hoc test. *** *p* ≤ 0.001; * *p* ≤ 0.05; ns—statistically non-significant (*p* > 0.05).

**Figure 6:**
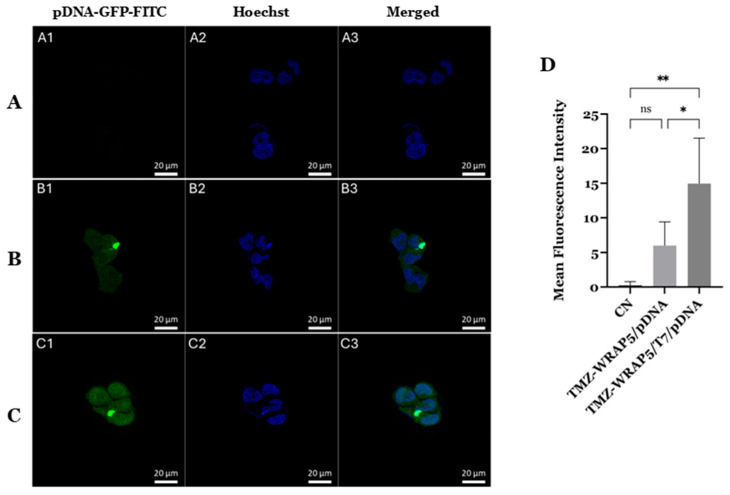
Confocal microscopy study (**A**–**C**) and quantification of Mean Fluorescence Intensity (MFI) using ImageJ software, version 1.54 (**D**). Representative confocal images of U87 MG cells after 4 h of transfection mediated by the different pDNA complexes developed at N/P ratio of 1. Hoechst dye stained the nuclei, whereas FITC-labeled pDNA was green. Panels (**A1**–**C1**) indicate FITC-labeled pDNA, panels (**A2**–**C2**) show nuclei stained with Hoechst, and panels (**A3**–**C3**) correspond to merged images. Non-transfected cells (**A**) and cells transfected with the developed complexes: TMZ-WRAP5/pDNA (**B**), TMZ-WRAP5/T7/pDNA (**C**). Scale bar = 20 µm. Statistical analysis: (**D**) one-way ANOVA followed by the Bonferroni post hoc test. ** *p* ≤ 0.01; * *p* ≤ 0.05; ns—statistically non-significant (*p* > 0.05).

**Figure 7:**
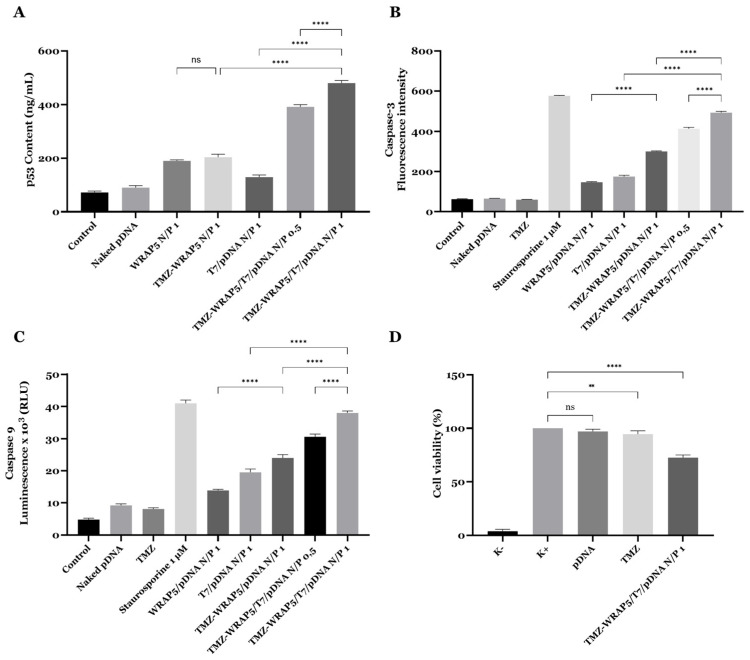
p53 expression and its therapeutic impact on U87 MG cells. p53 protein levels (**A**), caspase-3 (**B**), and caspase-9 activities (**C**) 24 h post-transfection mediated by peptide/pDNA complexes at N/P ratios of 0.5 and 1. In (**B**,**C**), staurosporine (1 µM) refers to the positive control, whereas untreated cells served as the negative control. Cellular viability (**D**) 72 h after transfection with peptide/pDNA complexes. U87 MG GB cells were transfected with TMZ-WRAP5/T7/pDNA at an N/P ratio of 1. Cells treated with ethanol were used as a negative control (K−), non-transfected cells as a positive control (K+), and cells treated with naked pDNA and TMZ were used as controls. Data were obtained from independent measurements (mean ± SD, n = 4 (**A**,**B**); n = 3 (**C**); n = 6 (**D**)) and analyzed by one-way ANOVA (**A**–**C**) or two-way ANOVA (**D**) followed by the Bonferroni post hoc test. **** *p* ≤ 0.0001; ** *p* ≤ 0.01; ns—non-significant (*p* > 0.05).

**Figure 8:**
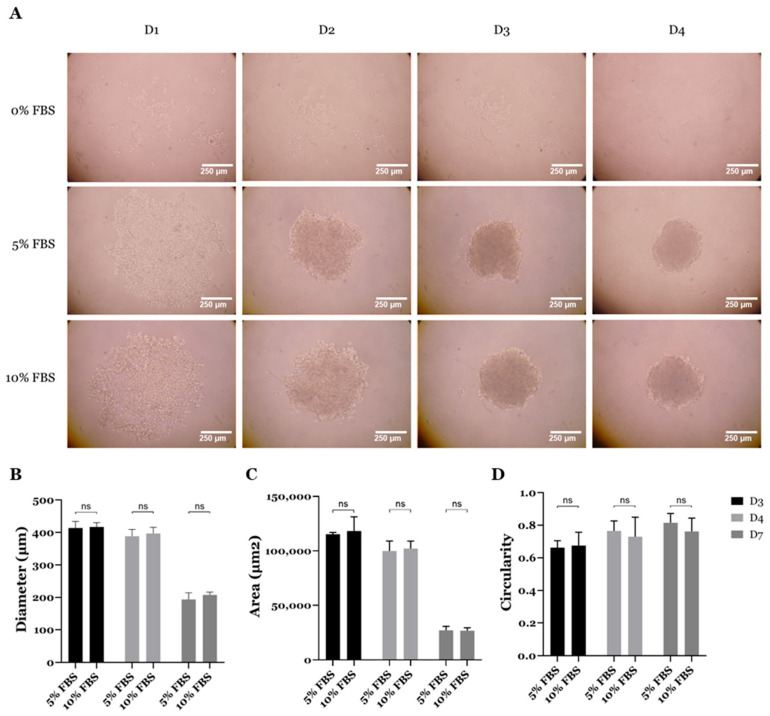
Morphological evolution (**A**) and quantitative analysis of U87 MG spheroid diameter (**B**), area (**C**), and circularity (**D**) cultured at different serum concentrations. Spheroids were formed from 7000 cells per well in agarose-coated 96-well flat-bottom plates using DMEM supplemented with 0%, 5%, or 10% FBS (*v*/*v*). Representative images acquired on days 1–4 post-seeding. Scale bar = 250 µm (**A**). Measurements were obtained at 3, 4, and 7 days of culture in medium supplemented with 5% or 10% FBS (**B**–**D**). Data represent SD (n = 4) from one independent experiment. Data were normalized to the respective day 3 control and analyzed by two-way ANOVA followed by the Bonferroni post hoc test. ns—not significant (*p* > 0.05).

**Figure 9:**
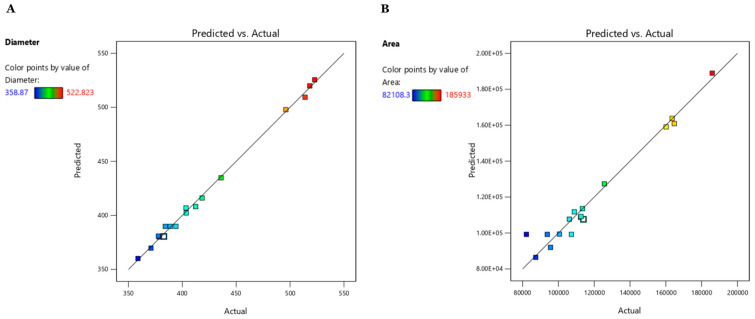
Predicted-versus-actual response plots. Spheroid diameter (**A**). Spheroid area (**B**). Data points represent individual experimental runs; the diagonal line represents perfect agreement between predicted and actual values.

**Figure 10:**
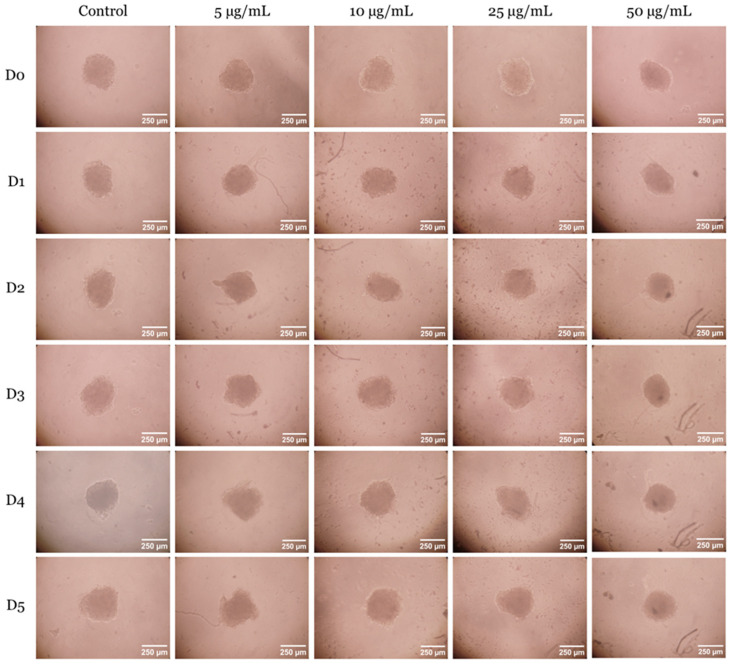
Morphological evolution of spheroid growth inhibition following TMZ-WRAP5/T7/pDNA nanocomplex treatment. Representative images of 3-day-old spheroids at (**D0**) (pre-transfection) and after 1, 2, 3, 4, and 5 days of transfection (**D1**–**D5**) exposed to nanocomplexes at N/P 1 with pDNA concentrations of 5, 10, 25, and 50 μg/mL. Untreated control spheroids shown for comparison. Scale bar = 250 μm.

**Figure 11:**
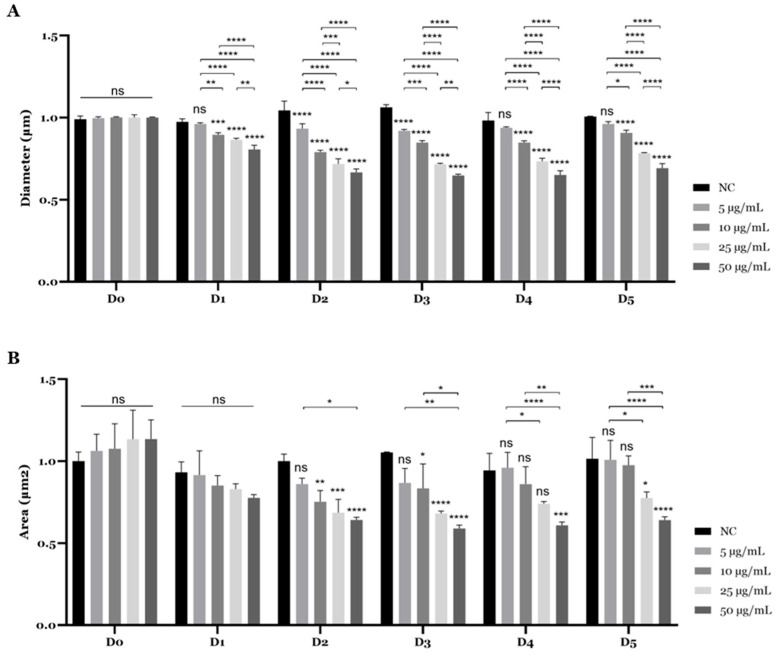
Quantitative analysis of spheroid growth inhibition following TMZ-WRAP5/T7/pDNA nanocomplex treatment. (**A**) Normalized spheroid diameter. (**B**) Normalized spheroid area. Data were normalized to the untreated control (NC) of D0 (pre-transfection) and represent mean ± SD (n = 3) from one independent experiment. Statistical analysis was performed using two-way ANOVA, followed by the Bonferroni post hoc test. **** *p* ≤ 0.0001; *** *p* ≤ 0.001; ** *p* ≤ 0.01; * *p* ≤ 0.05; ns—statistically non-significant (*p* > 0.05).

**Figure 12:**
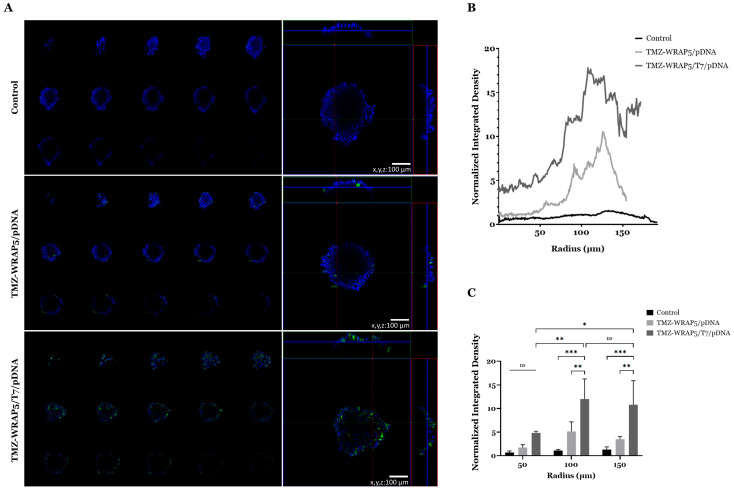
Penetration and uptake of peptide/pDNA nanocomplexes in U87 MG spheroids. (**A**) Representative z-stack images of spheroids 24 h post-transfection with FITC-labeled TMZ-WRAP5/pDNA and TMZ-WRAP5/T7/pDNA complexes (N/P 1; 50 µg/mL pDNA). Nuclei were counterstained with Hoechst 33342 (blue). Scale bar = 100 µm. (**B**) Radial profile analysis of normalized integrated intensity as a function of the spheroid radius (µm). (**C**) Quantitative comparison of normalized integrated intensity at specific radii (50, 100, and 150 µm). Data represent mean ± SD (n = 3) and were analyzed by two-way ANOVA followed by Bonferroni post hoc test. *** *p* ≤ 0.001; ** *p* ≤ 0.01; * *p* ≤ 0.05; ns—statistically non-significant (*p* > 0.05).

**Figure 13:**
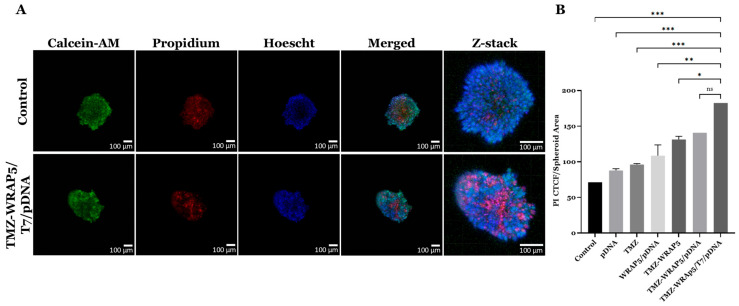
Live/Dead assessment of the therapeutic impact in U87 MG spheroids. (**A**) Representative confocal fluorescence microscopy images (SUM projection and z-stack reconstruction) of spheroids 72 h post-transfection. Viable cells are stained with Calcein-AM (green), dead cells with Propidium Iodide (PI, red), and the nuclei with Hoechst 33342 (blue). Scale bar = 100 µm. (**B**) Quantitative analysis of PI fluorescence intensity, corrected total cell fluorescence (CTCF) normalized to spheroid area, across all experimental groups. Data are expressed as mean ± SD (n = 3) and analyzed by one-way ANOVA followed by Bonferroni post hoc test. *** *p* ≤ 0.001; ** *p* ≤ 0.01; * *p* ≤ 0.05; ns—statistically non-significant (*p* > 0.05).

**Table 1:** The TMZ loading efficacy of TMZ-WRAP5/T7/pDNA complexes at varying N/P ratios.

System	N/P	[TMZ] Mean (µM)	Loading Rate %	
			Mean	SD
TMZ-WRAP5/T7/pDNA	N/P 0.5	288	88.7	0.18
	N/P 1	282	86.6	0.64

**Table 2:** Quadratic model summary statistics. Adeq—Adequate; SD—Standard Deviation.

Response	Source	SD	Sequential *p*-Value	Lack of Fit *p*-Value	R^2^	Adjusted R^2^	Predicted R^2^	Adeq Precision
Diameter	Quadratic	4.51	<0.0001	0.6459	0.9971	0.9934	0.9810	47.812
Area	Quadratic	8513.95	0.0005	0.9135	0.9655	0.9212	0.8918	15.7012

**Table 3:** DoE optimal point prediction and obtained mean (mean ± SD, n = 4). A—Agarose volume; B—Agarose percentage; C—Seeding cell number; PI—Prediction Interval; SD—Standard Deviation.

Predicted Input	Output	Predicted Mean	95% PI Low	95% PI High	Obtained Mean	SD
A-97.531 B-2.808% C-5441	Diameter	399.999	391.016	408.982	404.964	4.51
	Area	100,000	83,033.800	116,967	115,396	8513.95

## Data Availability

Data are contained within the article and Supplementary Materials.

## Supplementary Materials

The following supporting information can be downloaded at: https://www.mdpi.com/article/10.3390/ijms27156857/s1.

## Abbreviations

The following abbreviations are used in this manuscript:

2D	Two-Dimensional
3D	Three-Dimensional
AU	Arbitrary Units
BBB	Blood–brain barrier
CCD	Central Composite Designs
CCF	Face-Centered Composite
CPPs	Cell-Penetrating Peptides
CTCF	Corrected Total Cell Fluorescence
DLS	Dynamic Light Scattering
DNMTs	DNA Methyltransferases
DoE	Desing of Experiments
FBS	Fetal Bovine Serum
GB	Glioblastoma
HPLC	High-Performance Liquid Chromatography
IDH	Isocitrate Dehydrogenase
LOT	Liquid-Overlay Technique
MFI	Mean Fluorescence Intensity
MGMT	O6-methylguanine-DNA Methyltransferase
N/P	Nitrogen-to-Phosphate Groups
OFAT	One-Factor-At-a-Time
PdI	Polydispersity Index
PI	Propidium Iodide
RBCs	Red Blood Cells
SDS	Sodium Dodecyl Sulfate
SEM	Scanning Electron Microscopy
SM	Supplementary Materials
T7	HAIYPRH
TfR	Transferring Receptor
TMZ	Temozolomide
WHO	World Health Organization
