# Supplementary material for: Peptide-Based Nanocomplexes Enable Transferrin-Mediated Uptake and p53-Driven Antitumor Activity in 2D and 3D Glioblastoma Models
Source: Int J Mol Sci. 2026 Jul 30;27(15):6857. doi: 10.3390/ijms27156857 (PMC13466407; doi:10.3390/ijms27156857)
Supplement: Supplementary file 1 [file ijms-27-06857-s001.zip › ijms-4373075-supplementary.pdf]

Supplementary Materials

TMZ-WRAP5/T7/pDNA Complexation Behavior N/P Ratios of 0.1 and 0.2

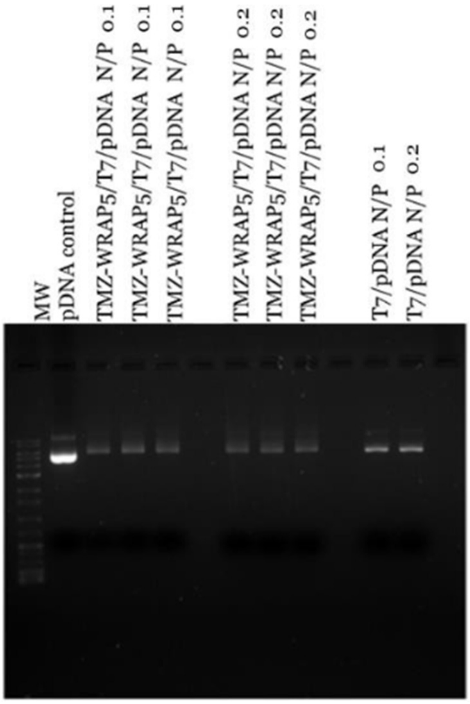

Figure S1. Electrophoretic analysis of TMZ-WRAP5/T7/pDNA complexes at N/P ratios 0.1 and 0.2. Agarose gel electrophoresis was performed to assess the complexation behavior and stability of pDNA in complexes formulated at N/P ratios of 0.1 and 0.2. After formulation and centrifugation, the supernatants were loaded on the gel for analysis. Lane 1: DNA molecular weight marker (MW); Lane 2: non-complexed pDNA; Lanes 3-5: TMZ/WRAP5/T7/pDNA at N/P 0.1; Lanes 7-9: TMZ/WRAP5/T7/pDNA at N/P 0.2; Lanes 11 and 12: T7/pDNA control complexes.

TMZ Complexation Efficiency

The standard curve was created using TMZ standards (Figure S2). These standards were prepared by diluting a stock solution of 10 mg/mL of TMZ in DMSO to various concentrations, ranging from 50 to 3200 µg/mL in ultrapure water. Absorbance measurements were taken at a wavelength of 330 nm using the NanoPhotometer™. The absorbance values of the TMZ standards were used to construct the standard curve, which was then applied to determine the drug concentrations of the unknown samples based on their absorbance values.

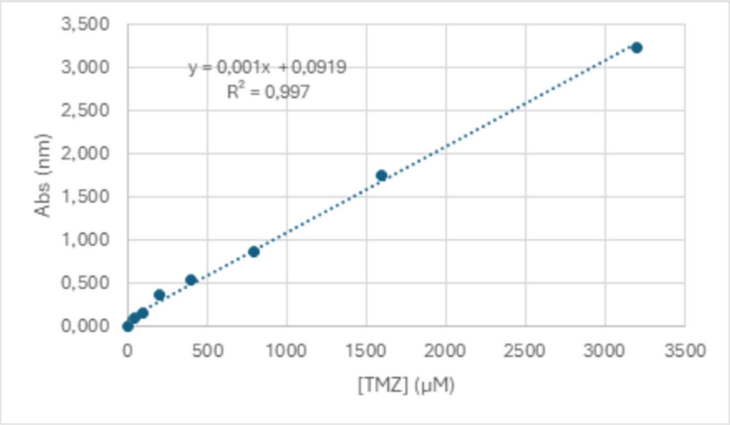

Figure S2. Standard curve of TMZ (reference standard) obtained by absorbance.

Peptide/pDNA Complexes Approach B (WRAP5/T7/TMZ/pDNA)

Two methods were evaluated for forming peptide/pDNA complexes. In Approach B, WRAP5/T7/TMZ/pDNA, formulation followed the same procedure as described for Approach A, but 50 µL of WRAP5/T7 solution was added to 150 µL of TMZ and pDNA (1 µg).

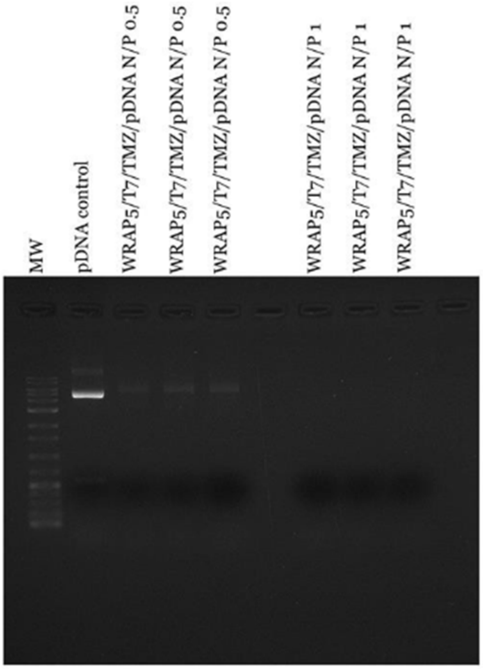

Figure S3. Electrophoretic analysis of WRAP5/T7/TMZ/pDNA complexes at different N/P ratios. Agarose gel electrophoresis was performed to assess the complexation behavior of pDNA in complexes formulated at N/P ratios of 0.5 and 1. After formulation and centrifugation, the supernatants were loaded on the gel for analysis. Lane 1: DNA molecular weight marker (MW); Lane 2: non-complexed pDNA; Lanes 3-5: WRAP5/T7/TMZ/pDNA at N/P 0.5; Lanes 7-9: WRAP5/T7/TMZ/pDNA at N/P 1.

Table S1. The TMZ loading rate of WRAP5/T7/TMZ/pDNA complexes at varying N/P ratios.

System	N/P	[TMZ] Mean (μM)	Loading Rate %	
			Mean	SD
**WRAP5/T7/TMZ/pDNA**	N/P 0.5	123	37.9	2.92
	N/P 1	127	39.1	2.68

Table S2. Physico-chemical properties of WRAP5/T7/TMZ/pDNA complexes at varying N/P ratios.

System	N/P	Size (nm)	PdI	Zeta Potential (mV)
**WRAP5/T7/TMZ/pDNA**	N/P 0.5	214 ± 4.45	0.409 ± 0.01	+6.11 ± 0.57
	N/P 1	201 ± 4.55	0.387 ± 0.01	+10.03 ± 0.91

Bax Quantification

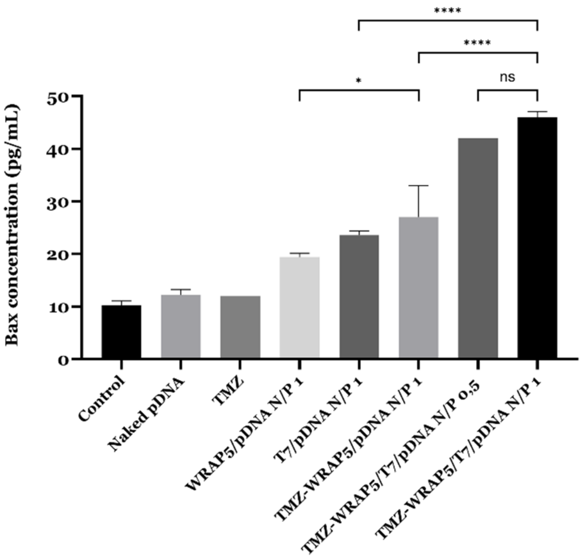

Figure S4. Quantitative assessment of Bax protein expression in U87 MG glioma cells. Bax protein levels were determined 24 h post-transfection mediated by peptide/pDNA nanocomplexes at N/P ratios of 0.5 and 1. Data were obtained from independent measurements (mean ± SD, n = 3) and analyzed by one-way ANOVA, followed by the Bonferroni test. **** $p \leq 0.0001$; * $p \leq 0.05$; ns – statistically non-significant ($p > 0.05$).

Design of Experiments for Model Optimization

Table S3. CCF experimental design and measured outputs for U87 MG spheroid formation optimization.

Run	Inputs			Outputs	
	Agarose (μL)	Agarose (%)	Cell Number	Diameter (μm)	Area (μm^2)
**1**	100	2	5,250	412.4	112,374.0
**2**	75	1	5,250	382.9	113,978.0
**3**	75	2	10,000	513.9	164,732.0
**4**	50	3	500	370.9	100,582.0
**5**	75	2	5,250	394.1	107,258.0
**6**	100	1	500	378.5	113,401.0
**7**	75	2	5,250	384.2	93,774.2
**8**	75	2	500	403.5	112,600.0
**9**	50	2	5,250	377.8	95,594.1
**10**	100	3	500	403.7	106,129.0
**11**	100	1	10,000	518.3	185,933.0
**12**	75	3	5,250	358.9	87,335.2
**13**	50	3	10,000	436.0	125,693.0
**14**	100	3	10,000	522.8	160,169.0
**15**	75	2	5,250	388.9	82,108.3
**16**	50	1	500	418.4	108,861.0
**17**	50	1	10,000	496.1	163,533.0

Table S4. ANOVA table for spheroid diameter and area responses. df – Degree of freedom.

Response	Source	Sum of Squares	df	Mean Square	F-value	p-value	Statistical output
**Diameter**	Model	49,476.92	9	5,497.44	270.53	< 0.0001	*significant*
	A-Volume	1,861.67	1	1,861.67	91.61	< 0.0001	
	B-%	1,039.30	1	1,039.30	51.14	0.0002	
	C-Cell number	26,233.65	1	26,233.65	1,290.97	< 0.0001	
	AB	2,355.35	1	2,355.35	115.91	< 0.0001	
	AC	1,688.59	1	1,688.59	83.10	< 0.0001	
	BC	138.92	1	138.92	6.84	0.0347	
	A^2	58.32	1	58.32	2.87	0.1341	
	B^2	1,022.63	1	1,022.63	50.32	0.0002	
	C^2	12,481.99	1	12,481.99	614.25	< 0.0001	
	Residual	142.25	7	20.32			
	Lack of Fit	93.90	5	18.78	0.7769	0.6459	*not significant*
	Pure Error	48.35	2	24.17			
**Area**	Model	1.421E+10	9	1.579E+09	21.79	0.0003	*significant*
	A-Volume	7.013E+08	1	7.013E+08	9.67	0.0171	
	B-%	1.119E+09	1	1.119E+09	15.44	0.0057	
	C-Cell number	6.681E+09	1	6.681E+09	92.17	< 0.0001	
	AB	2.140E+07	1	2.140E+07	0.2952	0.6038	
	AC	2.736E+08	1	2.736E+08	3.78	0.0931	
	BC	2.886E+08	1	2.886E+08	3.98	0.0862	
	A^2	3.368E+06	1	3.368E+06	0.0465	0.8355	
	B^2	1.304E+07	1	1.304E+07	0.1798	0.6842	
	C^2	3.435E+09	1	3.435E+09	47.38	0.0002	
	Residual	5.074E+08	7	7.249E+07			
	Lack of Fit	1.906E+08	5	3.812E+07	0.2407	0.9135	*not significant*
	Pure Error	3.168E+08	2	1.584E+08			

Table S5. Top 20 optimization solutions ranked by desirability.

Number	Volume	%	Cell Number	Diameter	Area	Desirability
1[1]	97.531	2.808	5,441.443	399.999	100,000.159	1.000
2	97.282	2.849	5,546.157	400.000	99,999.800	1.000
3	97.287	2.987	5,824.238	400.000	100,000.142	1.000
4	98.301	2.742	5,236.925	400.000	99,999.936	1.000
5	97.146	2.909	5,678.498	400.000	100,000.131	1.000
6	97.233	2.862	5,576.279	400.001	99,999.951	1.000
7	98.127	2.754	5,276.748	399.999	99,999.962	1.000
8	97.145	2.922	5,705.500	399.999	100,000.083	1.000
9	97.319	2.994	5,837.552	400.000	99,999.868	1.000
10	97.195	2.874	5,602.883	400.000	100,000.146	1.000
11	99.531	2.682	4,994.736	400.001	99,999.854	1.000
12	97.150	2.902	5,663.570	400.000	100,000.104	1.000
13	97.262	2.979	5,811.745	400.000	99,999.904	1.000
14	99.803	2.672	4,945.887	400.000	99,999.883	1.000
15	97.159	2.893	5,645.563	400.000	99,999.997	1.000
16	98.633	2.723	5,165.939	400.000	99,999.982	1.000
17	97.315	2.842	5,528.617	400.000	100,000.179	1.000
18	95.932	2.999	5,984.990	400.000	100,302.610	0.998
19	97.565	3.000	5,849.297	400.404	100,130.185	0.998
20	50.001	1.703	1,491.048	399.999	100,588.785	0.997

[1] Selected solution for experimental validation.

Live/Dead Spheroid Assay

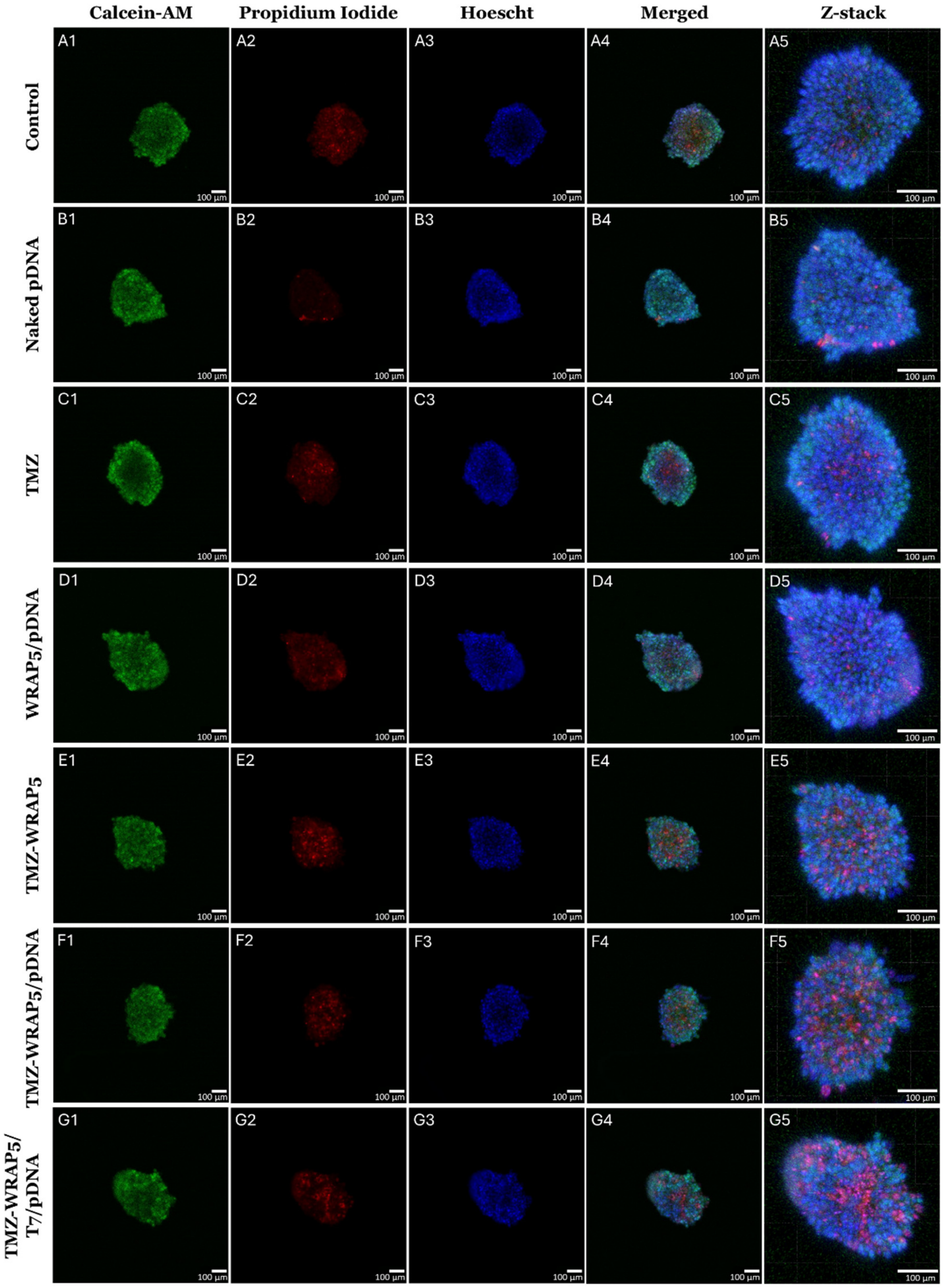

Figure S5. Live/Dead assessment in U87 MG spheroids. (**A**) Representative confocal fluorescence microscopy images (SUM projection and z-stack reconstruction) of spheroids 72 h post-transfection. Viable cells are stained with Calcein-AM (green), dead cells with Propidium Iodide (PI, red), and the nuclei with Hoechst 33342 (blue). Scale bar = 100 µm.
